# Multivariate Analysis of Grain Yield and Its Attributing Traits in Different Maize Hybrids Grown under Heat and Drought Stress

**DOI:** 10.1155/2015/563869

**Published:** 2015-12-20

**Authors:** Fawad Ali, Naila Kanwal, Muhammmad Ahsan, Qurban Ali, Irshad Bibi, Nabeel Khan Niazi

**Affiliations:** ^1^Department of Plant Breeding and Genetics, University of Agriculture Faisalabad, Faisalabad 38040, Pakistan; ^2^Centre of Excellence in Molecular Biology, University of the Punjab, Lahore 54590, Pakistan; ^3^Institute of Soil and Environmental Sciences, University of Agriculture Faisalabad, Faisalabad 38040, Pakistan

## Abstract

This study was carried out to evaluate F1 single cross-maize hybrids in four crop growing seasons (2010–2012). Morphological traits and physiological parameters of twelve maize hybrids were evaluated (i) to construct seed yield equation and (ii) to determine grain yield attributing traits of well-performing maize genotype using a previously unexplored method of two-way hierarchical clustering. In seed yield predicting equation photosynthetic rate contributed the highest variation (46%). Principal component analysis data showed that investigated traits contributed up to 90.55% variation in dependent structure. From factor analysis, we found that factor 1 contributed 49.6% variation (*P* < 0.05) with primary important traits (i.e., number of leaves per plant, plant height, stem diameter, fresh leaves weight, leaf area, stomata conductance, substomata CO_2_ absorption rate, and photosynthetic rate). The results of two-way hierarchical clustering demonstrated that Cluster III had outperforming genotype H_12_ (Sultan × Soneri) along with its most closely related traits (photosynthetic rate, stomata conductance, substomata CO_2_ absorption rate, chlorophyll contents, leaf area, and fresh stem weight). Our data shows that H_12_ (Sultan × Soneri) possessed the highest grain yield per plant under environmentally stress conditions, which are most likely to exist in arid and semiarid climatic conditions, such as in Pakistan.

## 1. Introduction

Maize (*Zea mays *L.) is one of the most commonly cultivated crop worldwide [1, 2]. Heat and drought stress have emerged as a common problem worldwide which can reduce maize crop productivity [3]. The right choice of maize genotypes for a given region is a crucially important practice to obtain high grain yield of different maize hybrids [4]. Quantitative evaluation of traits in the field experiments is dependent on soil heterogeneity [5], genetic variability of the experimental material [6], and biotic and abiotic factors [7]. In addition to heat and drought stress, intraseasonal and interseasonal water availability variation are another common and significant problem that can lead to decrease grain yield of maize hybrids in various regions of the world [8]. Several studies have been conducted to understand the maize plant phenology (plant development rate) and physiology (functioning of internal processes) in different seasons and regions [9]. It is a well-known fact that high temperature stress and low irrigation regimes can limit certain factors in maize plant, thereby decreasing plant biomass below the target (7.5 tons/acre) levels [10, 11].

Considering these important, although partially resolved, research aspects, we conducted a field study in four consecutive crop growing seasons (2010-2011 and 2011-2012). The objective of the present study was to demonstrate combined effect of seasonal variation on maize grain yield and its attributing traits in maize hybrids under high temperature (>45°C) and drought stress conditions. The two-way hierarchical clustering was used for making clusters within and among the genotypes and traits for effective selection. This allowed us to develop a model for predicting grain yield per plant thus selecting the most promising traits of high yielding maize hybrids under investigated stress environments.

## 2. Materials and Methods 

### 2.1. Experimental Details

The four maize inbred lines: Agaiti-85 (P_1_)_,_ Golden (P_2_), Soneri (P_3_) and Sultan (P_4_) from F_7_ population (99.25% purity) under high temperature (>45°C) and drought stress conditions were selected during 2009-2010 in the University of Agriculture, Faisalabad, Pakistan (31°26′N, 73°06′E). Two irrigation regimes were applied to each plot fortnightly. The experimental sites have been managed for more than 50 years under continuous agriculture. The textural class of soil was clay loam and the soil pH was 7.81 (soil : water, 1 : 5). The average organic matter content in the soil was 1.76%, which was relatively higher than average organic matter content, due to addition of wheat stubbles every year at the experimental site.

### 2.2. Genetic Material

Parental lines were crossed in a complete Diallel fashion to develop F_1_ single cross hybrids. The maize hybrids were H_1_ (Agaiti-85 × Golden), H_2_ (Agaiti-85 × Soneri), H_3_ (Agaiti-85 × Sultan), H_4_ (Golden × Agaiti-85), H_5_ (Golden × Soneri), H_6_ (Golden × Sultan), H_7_ (Soneri × Agaiti-85), H_8_ (Soneri × Golden), H_9_ (Soneri × Sultan), H_10_ (Sultan × Agaiti-85), H_11_ (Sultan × Golden), and H_12_ (Sultan × Soneri).

Maize plants were cultivated in the field in a randomized complete block design (RCBD) with five replicates per genotype. Hybrids were evaluated for grain yield in four consecutive crop growing seasons: 2011 and 2012 (February and August). Combined data was used for statistical analysis to reduce effect of crop growing seasons to optimize grain yield and its attributing traits in either of crop growing seasons.

Each plot was of 3 m × 3 m size. Row-to-row and plant-to-plant distances were 75 cm and 15 cm, respectively, with each row having 15 plants. Seed sowing was done using a dibbler. Two seeds/hill of each genotype were sown and after 20 days thinned up to one plant/hill. All recommended cultural and agronomic practices such as howing, mowing, irrigation, fertilizer application, and weeding were done during crop growing seasons.

Random sampling of 10 plants/plot of each genotype was done to measure the following traits: chlorophyll content (Ch.c.), plant height (PH), stem diameter (SD), fresh stem weight (FSW), number of leaves per plant (nlp), fresh leaves weight (FLW), fresh leaves weight to stem weight ratio (FLSWR), leaf area (LA), leaf length (LL) from acute angle, leaf width (LW), transpiration rate (*E*), leaf temperature (LT), photosynthetic rate (*A*), stomata conductance (*g*_*s*_), substomata CO_2_ absorption rate (*C*_*i*_), and grain yield (GY) per plant. Grain yield was recorded in kilogram per hectare.

### 2.3. Phenological Data of Maize Crop

The data of different crop growth stages such as seedling emergence, silking and physiological maturity of maize plants were recorded for each experimental plot.

### 2.4. Green Fodder Yield and Its Partitioning

Plant height, leaf length, and leaf width were measured using a measuring tape. The fresh leaf weight and fresh stem weight were recorded. Digital vernier caliper was used to measure stem diameter by computing average values of measured stem diameters at basal, middle, and top portions.

### 2.5. Physiological Parameters: Radiation Capture, Leaf Temperature, and CO_2_ Absorption

Green leaf area per plant was determined according to *L* × *W* × 0.75 [12]. Infrared Radiation Gas Analyzer (IRGA, LCi Photosynthetic System, ADC Bio Scientific Ltd., software version 2) was used on a daily basis at late morning, noon, and early afternoon times, up to booting stage during the four consecutive crop growing seasons. This allowed us to measure the physiological parameters photosynthetic rate (*A*), transpiration rate (*E*), stomata conductance, (*g*_*s*_), substomata CO_2_ absorption rate (*C*_*i*_), and leaf temperature (LT). SPAD chlorophyll meter was used to measure chlorophyll contents in leaves.

### 2.6. Grain Yield

At harvesting, 10 plants of each genotype were sampled. Cobs from plants of each genotype were harvested to record the grain yield per plant. The moisture level of the grains was adjusted (14–15.5%) to 140 g/kg [13] for future breeding program.

### 2.7. Statistical Procedures

A two-way analysis of variance (ANOVA) was used to determine significant differences for grain yield and its attributing traits. The stepwise multiple linear regression was performed between the seed yield and its attributing traits to construct seed yield equation. GenStat version 12 software was used for statistical analysis of data. Multivariate analysis was performed (PROC Mixed SAS version 9.1, SAS Institute [14]) for principal component analysis, factor analysis, and two-way hierarchical clustering.

## 3. Results and Discussion

In the present study, analysis of variance of all studied traits in maize hybrids (Table S1, in Supplementary Material available online at http://dx.doi.org/10.1155/2015/563869) showed significant differences (^*∗*^ *P* < 0.01). For parental genotypes (Sultan and Soneri), DMRT was found to be significant (^*∗*^ *P* < 0.01) for grain yield per plant (837 and 812 g/m^2^, resp.) as shown in Table S2. Among all studied maize hybrids, H_12_ (Sultan × Soneri) was found to be significant (^*∗*^ *P* < 0.01) for the highest grain yield (1781 g/m^2^) (Supplementary Material, Table S3).

For hybrid H_12_ *g*_*s*_, *A*, Ch.c., and SD traits had the significant mean values (^*∗*^ *P* < 0.01). The previous studies [4, 15, 16] showed that DMRT provided a significant importance to differentiate the pair of means while examining the effect of morphophysiological and agronomic traits on grain yield in maize hybrids. To predict seed yield equation in maize hybrids [17–19], we used a stepwise regression model. It was found that photosynthetic rate contributed a large variation (46%) while *R* ^2^ value for all traits (Table 1) was 74%.

The best prediction equation for grain yield in the present study was as follows:

(1) Y=22.23+−0.035X1+−0.049X2+0.007X3+−0.121X4+−0.066X5+−0.001X6+−0.062X7+−0.012X8+0.130X9+−0.015X10+0.026X11+1.320X12+−0.379X13+0.002X14+−0.013X15.

We showed that photosynthetic rate contributed the maximum variation (Table 1) in seed yield predicted equation but it could be biased as previous literature also reported the error effect of stepwise regression [19] while handling a large number of independent variables. Cirilo et al. [13] used PCA to overcome the effect of large number of independent variables in breeding experiments and find overall attributed variation in dependent structure. Similarly, Greenacre [20] reported that eigenvalues (in PCA) have primary importance for numerical diagnostics to assess variation attributed by number of large variables on the dependent structure and their data matrix in a graphical display.

**Figure 1:**
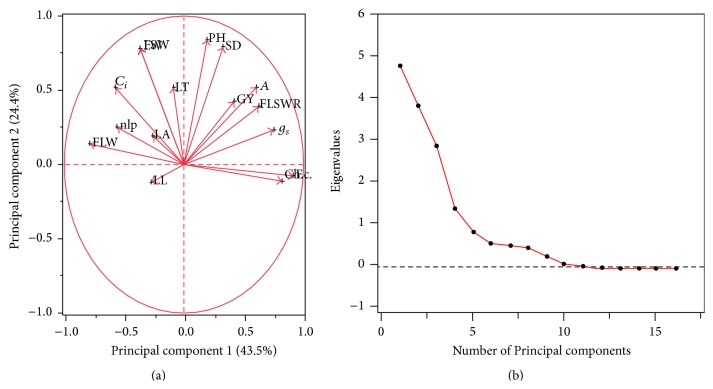
(a) Principal component analysis of grain yield and its attributing traits. (b) Scree plot and respective eigenvalues (see Section 2 for hybrid codes and traits description).

A PCA was performed using various traits under investigation (Supplementary Material, Table S4) and three principal components (PCs) were observed: PC1, PC2, and PC3 (Figure 1(a)). Their eigenvalues were more than 1 (Figure 1(b)) and PC1, PC2, and PC3 contributed variations of 72%, 28%, and 10%, respectively. Cumulatively these three PCs contributed 89.6% of total variation to grain yield per plant. Based on PCA, we performed factor analysis (FA) to determine the latent factors or groups of variables (Table 2 and Table S5 (Supplementary Material)). The variables included in the first factor were “*g*_*s*_, *A*, *C*_*i*_, nlp, PH, FLW, FLSWR, and LA” which explained 49.85% of total variance in grain yield per plant thus showed high importance for primary selection in under-study maize hybrids.

**Figure 2:**
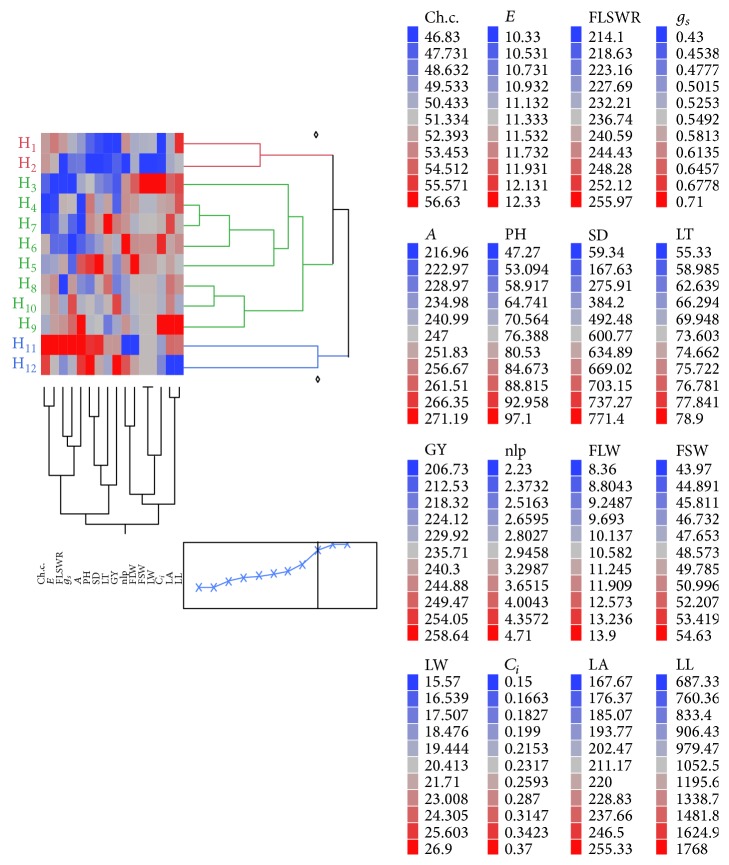
Dendrogram analysis based on two-way hierarchal clustering. Association of hybrids and traits based on genetic basis (see Section 2 for hybrid codes and traits description).

Our findings are well supported by Filipović et al. [21] who demonstrated the role of factor analysis for effective selection criteria in maize breeding program. Moreover, making groups or clusters of under-study maize genotypes is an efficient tool to minimize the plant pool during selection process [6, 22, 23]. The previous literature described the use of one-way cluster analysis (intrarelationship) but lacks information about effective grouping between hybrids and traits as we did in this study. The two-way hierarchical clustering was employed to explore the possible intra- and interrelationships among hybrids and traits at the same time. This relationship allowed us to develop new and most effective way of desired traits selection for breeding high yielding cultivars (H_12_) in arid/semiarid zone (Figure 2). The two-way hierarchical clustering demonstrated relationship of “traits-specific” to genotype relationship based on hierarchy. In the present study, we obtained three clusters including Cluster I, Cluster II, and Cluster III. It was found that Cluster III had genotype H_12_ (Sultan × Soneri) with its closely related morphophysiological traits (*A*, *g*_*s*_, *C*_*i*_, Ch.c., LA, and FSW) (Figure 2). This indicated that, in future breeding program of H_12_, these traits are important for primary selection to increase grain yield under high temperature stress and low irrigation regimes. Moreover, this method proved to be more efficient as it reduced the cost, money, time, and efficacy for better selection in maize crop improvement program. However, further studies are required which should cover different years and locations.

## 4. Conclusions

The present study provided insights into drought tolerant and heat resistant maize hybrids for arid/semiarid regions like Pakistan. A new way of two-way hierarchical clustering was used which enabled us to develop a relationship among the hybrids and morphophysiological traits. We used a combination of physiological strategies and breeding methods to evaluate the maize hybrids in four consecutive crop growing seasons over a period of two years (2010-2011 and 2011-2012). The results showed that H_12_ possessed the highest grain yield under high temperature stress and low irrigation regime. Our findings also provide insights to understand GT factors, which are considered to be valuable for future breeding programs in maize. However, further research is warranted on different locations and climatic conditions.

## Supplementary Material

(Suppl. Table S1). Mean Square values of Analysis of variance (Combined over both seasons) of yield and its attributing traits in maize [see section materials and methods for parental codes and traits description] .
(Suppl. Table S2). Duncan Multiple Range Test (DMRT) for grain yield and its components of four parental lines [see section materials and methods for hybrids and traits description] .
(Suppl. Table S3). Duncan Multiple Range Test (DMRT) for grain yield and yield attributing traits of twelve maize hybrids (combined over both seasons) [see section materials and methods for hybrids and traits description].
Suppl. Table S4. Eigen vectors (loadings) of first three principal components of yield and yield attributing traits [ see section materials and methods for traits description ].
Suppl. Table S5. Principal factor matrix after rotated factor loadings for yield and yield attributing traits [ see section materials and methods for traits description ].

## Figures and Tables

**Table 1:** Stepwise multiple linear regression of grain yield attributing traits (see Section 2 for traits description).

	Variable	Regression coefficients		*T*	Cumulative *R* ^2^	Partial *R* ^2^ (%)
		*B*	SE (±)			
*X*_1_	*A*	−0.03532	0.01436	1.99	0.4692	46.9%
*X*_2_	*g*_*s*_	−0.04996	0.03239	1.33	0.3340	33.4%
*X*_3_	Ch.c.	0.00797	0.00200	1.20	0.3044	30.4%
*X*_4_	FSW	−0.12108	0.01756	−1.06	−0.2717	27.1%
*X*_5_	nlp	−0.06664	0.02666	−1.00	−0.2581	25.8%
*X*_6_	SD	0.00152	1.523*E* − 04	−0.99	−0.2568	25.6%
*X*_7_	*C*_*i*_	−0.06247	0.00797	0.98	0.2544	25.4%
*X*_8_	LW	−0.01236	0.00385	−0.93	−0.2418	24.1%
*X*_9_	PH	0.13092	0.01899	0.57	0.1515	15.1%
*X*_10_	FLSWR	−0.01528	0.00358	−0.49	−0.1306	13.1%
*X*_11_	LA	0.02657	0.00494	0.44	0.1179	11.7%
*X*_12_	LL	1.32010	0.20570	−0.33	−0.0870	8.7%
*X*_13_	*E*	−0.37968	0.07671	0.28	0.0733	7.3%
*X*_14_	FLW	0.00241	4.012*E* − 04	−0.22	−0.0588	5.8%
*X*_15_	LT	−0.01345	0.01183	0.03	0.0080	0.8%

Intercept = −22.23. Multiple *R* = 0.87 (87%). *R* ^2^ = 0.74 (74%). Adjusted *R* ^2^ = 0.73 (73%). Standard error (SE) of estimation = 5.12.

**Table 2:** Factor loadings of grain yield attributing morphophysiological and agronomic traits (see Section 2 for traits description).

Variables	Loadings	% of total communality
Factor 1		49.85
nlp	0.818	
PH	0.824	
SD	0.502	
FLW	0.790	
LA	0.764	
*C*_*i*_	0.866	
*A*	0.759	
*g*_*s*_	0.803	

Factor 2		29.47
Ch.c.	−0.749	
*E*	−0.899	
FLSWR	−0.950	

Factor 3		11.22
LL	0.373	
LT	0.171	
Cumulative variance		90.55

## Abbreviations

DMRT: Duncan's Multiple Range Test
PCA: Principal component analysis
FA: Factor analysis.
