# Supplementary material for: Multivariate Analysis of Grain Yield and Its Attributing Traits in Different Maize Hybrids Grown under Heat and Drought Stress
Source: Scientifica (Cairo). 2015 Dec 20;2015:563869. doi: 10.1155/2015/563869 (PMC4699226; doi:10.1155/2015/563869)
Supplement: Supplementary file 1 — (Suppl. Table S1). Mean Square values of Analysis of variance (Combined over both seasons) of yield and its attributing traits in maize [see section materials and methods for parental codes and traits description] . (Suppl. Table S2). Duncan Multiple Range Test (DMRT) for grain yield and its components of four parental lines [see section materials and methods for hybrids and traits description] . (Suppl. Table S3). Duncan Multiple Range Test (DMRT) for grain yield and yield attributing traits of twelve maize hybrids (combined over both seasons) [see section materials and methods for hybrids and traits description]. Suppl. Table S4. Eigen vectors (loadings) of first three principal components of yield and yield attributing traits [ see section materials and methods for traits description ]. Suppl. Table S5. Principal factor matrix after rotated factor loadings for yield and yield attributing traits [ see section materials and methods for traits description ]. [file 563869.f1.pdf]

Supplementary Tables

(Suppl. Table S1). Mean Square values of Analysis of variance (Combined over both seasons) of yield and its attributing traits in maize [see section materials and methods for parental codes and traits description]

SOV	Ch.	P.H	S.D	FLW	L.A	L.W	FS.W	LL
**Parents at maturity**	11.71*	286.57*	0.004**	6228**	24814.26**	410.03**	3559.1**	0.21*
**F1 at maturity**	23.38	548.85*	0.009*	213.06*	8646.4**	594.6**	4180.6**	1.34**

SOV	FLSWR	A	gs	E	L.T	Ci	nlp	GY
**Parents at maturity**	0.19*	177.3**	0.04**	44.3**	12.16*	16372.8**	4.33*	1191.1*
**F1 at maturity**	0.23**	80.13**	0.013*	8.012**	33.08**	2545.1**	1.29*	14239.53*

* = **Significance at (*P < 0.05)**
= **Significance at (*P < 0.01)

(Suppl. Table S2). Duncan Multiple Range Test (DMRT) for grain yield and its components of four parental lines [see section materials and methods for hybrids and traits description]

Ch.C		P.H		SD		LA	
μg/L		cm		cm		m^2	
Genotypes	DMRT	Genotypes	DMRT	Genotypes	DMRT	Genotypes	DMRT
P4	45.82^{A}	P4	78.44^{A}	P4	0.80^{A}	P4	433.89^{A}
P2	44.58^{A}	P3	76.69^{A}	P3	0.73^{B}	P3	420.16^{A}
P3	43.70^{AB}	P1	66.42^{B}	P2	0.71^{B}	P1	383.70^{A}
P1	40.74^{B}	P2	63.04^{C}	P1	0.56^{C}	P2	278.21^{B}
FSW		A		gs		E	
g		μmol/m^2s		mmol/m^2s		mm/s	
Genotypes	DMRT	Genotypes	DMRT	Genotypes	DMRT	Genotypes	DMRT
P4	139.68^{A}	P3	23.73^{A}	P4	0.32^{A}	P2	14.13^{A}
P3	96.78^{B}	P4	17.68^{B}	P2	0.21^{B}	P1	11.38^{B}
P2	86.06^{C}	P2	17.68^{B}	P3	0.14^{C}	P3	8.24^{C}
P1	81.04^{C}	P1	9.24^{C}	P1	0.11^{C}	P4	7.75^{C}
LT		SSC/Ci		Nlp		FLW	
^{o}C		vpm				g	
Genotypes	DMRT	Genotypes	Genotypes	Genotypes	DMRT	Genotypes	54.98^{A}
P1	48.02^{A}	P4	P4	P4	13.61^{A}	P4	46.90^{B}
P2	47.56^{A}	P3	P3	P3	12.82^{AB}	P3	39.19^{C}
P3	47.28^{A}	P1	P1	P1	12.21^{AB}	P1	34.31^{C}
P4	44.56^{B}	P2	P2	P2	11.42^{B}	P2	54.98^{A}
LL		LW		FLSWR		GY	
Cm		Cm				g	
Genotypes	DMRT	Genotypes	DMRT	Genotypes	DMRT	Genotypes	DMRT
P4	67.10^{A}	P4	6.56	P4	2.54	P4	837.60^{A}
P3	66.68^{AB}	P3	6.41	P3	2.41	P3	812.00^{AB}
P1	58.61^{BC}	P1	6.26	P2	2.20	P2	654.20^{BC}
P2	52.12^{C}	P2	4.74	P1	2.01	P1	568^{D}

Values in the same column with the same letter are not significantly different (*P < 0.01).

(Table S3). Duncan Multiple Range Test (DMRT) for grain yield and yield attributing traits of twelve maize hybrids (combined over both seasons) [see section materials and methods for hybrids and traits description]

CH.C		P.H		SD		LA	
		cm		cm		m^2	
Hybrids	DMRT	Hybrids	DMRT	Hybrids	DMRT	Hybrids	DMRT
H12	56.633^{A}	H12	255.97^{A}	H12	0.71^{A}	H12	733.85^{A}
H11	55.367^{AB}	H11	252.27^{AB}	H11	0.61^{B}	H7	729.91^{A}
H9	55.233^{AB}	H10	249.63^{AB}	H10	0.60^{B}	H11	693.51^{AB}

H8	53.033^{ABC}	H9	245.97^{ABC}	H9	0.60^{B}	H8	658.87^{ABC}
H7	52.867^{ABC}	H7	243.90^{ABC}	H7	0.60^{B}	H1	648.56 ^{ABC}
H10	52.733^{ABC}	H3	238.10^{ABCD}	H6	0.59^{B}	H9	647.60 ^{ABC}
H1	52.433^{ABC}	H2	238.03^{ABCD}	H8	0.57^{B}	H5	640.16 ^{ABC}
H6	52.033^{ABC}	H4	237.63^{ABCD}	H3	0.57^{B}	H2	627.79^{BC}
H4	51.633^{ABC}	H8	236.80^{ABCD}	H5	0.57^{B}	H6	626.76 ^{BC}
H5	50.600^{ABC}	H6	234.67^{ABCD}	H4	0.56^{B}	H4	621.93 ^{BC}
H2	50.433^{ABC}	H5	230.40^{ABCD}	H3	0.55^{BC}	H2	593.94^{C}
H3	49.833^{BC}	H2	224.07^{BCD}	H1	0.55^{BC}	H3	581.39 ^{C}
FLW<br>G		FSW<br>g		A<br>μmol/m^{2}s		gs<br>mmol/m^{2}s	
Hybrids	DMRT	Hybrids	DMRT	Hybrids	DMRT	Hybrids	DMRT
H12	106.57^{A}	H12	328.13^{A}	H12	27.23^{A}	H12	0.36^{A}
H11	97.10^{AB}	H11	244.33^{B}	H11	26.90^{A}	H10	0.35^{A}
H8	89.27^{ABC}	H10	244.30^{B}	H8	26.60^{A}	H11	0.32^{AB}
H7	88.90 ^{ABC}	H8	243.17^{B}	H10	25.70^{AB}	H8	0.30^{ABC}
H10	83.73 ^{ABC}	H7	231.47^{B}	H7	25.10^{AB}	H6	0.30^{ABC}
H9	81.77 ^{ABC}	H9	215.70^{B}	H6	24.70^{ABC}	H7	0.26^{ABCD}
H6	80.90 ^{ABC}	H5	214.73^{B}	H9	23.90^{ABCD}	H9	0.23^{ABCD}
H5	77.90^{BC}	H2	209.43^{BC}	H5	19.73^{BCDE}	H5	0.22^{ABCD}
H2	73.70^{BCD}	H6	207.97^{BC}	H2	19.30^{BCDE}	H1	0.22^{ABCD}
H1	72.37 ^{BCD}	H1	207.77^{BC}	H1	18.65^{CDE}	H3	0.18^{BCD}
H4	71.37 ^{BCD}	H4	198.87^{BC}	H4	18.10^{DE}	H2	0.17^{BCD}
H3	70.70 ^{BCD}	H3	197.70^{BC}	H4	17.47^{E}	H4	0.16^{BCD}
E<br>mm/s		LT<br>^{o}C		SSC/Ci<br>vpm		nlp	
Hybrids	DMRT	Hybrids	DMRT	Hybrids	DMRT	Hybrids	DMRT
H6	13.90^{A}	H9	54.63^{A}	H12	266.33^{A}	H12	12.66^{A}
H9	12.13^{AB}	H1	51.86^{AB}	H11	255.33^{AB}	H11	12.01^{AB}
H7	11.93^{AB}	H5	50.36^{ABC}	H1	249.67^{AB}	H9	12.01^{AB}
H8	11.76^{ABC}	H10	50.26^{ABC}	H7	248.67^{AB}	H10	12.01^{AB}
H10	11.73^{ABC}	H7	49.93^{ABCD}	H8	223.33^{ABC}	H5	11.66^{ABC}
H5	11.46^{ABCD}	H8	49.76^{ABCDE}	H9	216.00^{ABC}	H8	11.66^{ABC}
H2	11.40^{ABCD}	H4	49.10^{ABCDE}	H6	213.00^{ABC}	H6	11.66^{ABC}
H4	10.95^{BCDE}	H6	48.20^{ABCDE}	H10	210.67^{ABC}	H4	11.33^{ABCD}
H11	10.76^{BCDE}	H2	47.40^{BCDE}	H2	204.33^{BC}	H7	11.33^{ABCD}
H12	10.01^{BCDE}	H3	45.96^{BCDE}	H3	204.00^{BC}	H2	11.33^{ABCD}
H3	9.27^{BCDE}	H11	45.76^{BCDE}	H5	201.00^{BC}	H1	11.33^{ABCD}
H1	8.98^{CDE}	H12	45.63^{BCDE}	H4	197.33^{BC}	H3	11.01^{BCD}
LW		LL		FLSWR		G.Y	

Cm		Cm				g	
Hybrids	DMRT	Hybrids	DMRT	Hybrids	DMRT	Hybrids	DMRT
H12	8.96A	H12	82.53^{A}	H12	4.71^{A}	H12	1768.7^{A}
H11	8.93 A	H11	80.10^{AB}	H11	3.43^{B}	H11	1581.7^{AB}
H10	8.90A	H10	78.00^{B}	H10	3.58^{B}	H10	1380.0^{BC}
H6	8.63AB	H6	78.80^{B}	H8	3.43^{B}	H7	1266.0^{BC}
H8	8.63AB	H8	77.13^{BC}	H7	3.24^{BC}	H6	1066.0^{BCD}
H9	8.56AB	H9	77.47^{BC}	H9	3.14^{BC}	H9	991.0^{BCD}
H4	8.50ABC	H4	76.90^{BCD}	H5	2.89^{CD}	H5	958.7^{BCD}
H1	8.46ABCD	H1	75.53^{BCDE}	H2	2.79^{CD}	H8	874.0^{BCD}
H7	8.46ABCD	H7	75.20^{BCDE}	H6	2.78^{CD}	H2	840.0^{BCD}
H3	8.43ABCD	H3	74.20^{BCDE}	H1	2.77^{CD}	H4	768.7^{BCD}
H4	7.93BCD	H4	74.43^{BCDE}	H4	2.43^{CDE}	H3	767.0^{BCD}
H2	7.83BCD	H2	72.90^{CDEF}	H3	2.36^{CDE}	H1	748.3^{BCD}

Values in the same column with the same letter are not significantly different (*P < 0.01)

Suppl. Table S4. Eigen vectors (loadings) of first three principal components of yield and yield attributing traits [see section materials and methods for traits description].

Traits	PCA1	PCA2	PCA3
Ch.c	0.951	-0.118	0.005
nlp	0.957	-0.211	0.032
PH	0.928	0.082	-0.168
SD	0.832	0.354	0.170
FSW	0.653	0.422	0.603
FLW	0.975	-0.078	0.049
FLSWR	-0.809	0.341	0.392
LL	0.490	-0.729	0.403
LW	0.922	0.164	-0.097
LA	0.935	-0.117	0.056
E	-0.938	0.096	0.210
LT	0.251	0.865	-0.353
A	-0.912	-0.016	0.038
gs	-0.920	0.150	0.028
Ci	0.747	-0.152	-0.418
GY	0.851	0.259	0.426

Suppl. Table S5. Principal factor matrix after rotated factor loadings for yield and yield attributing traits [see section materials and methods for traits description].

Variables	Factors			Final Communality Estimates (h^2)
	Factor 1	Factor 2	Factor 3	
Ch.c	-0.749	-0.595	-0.052	0.918
nlp	0.818	0.480	-0.250	0.962
PH	0.824	0.454	0.104	0.895
SD	0.502	0.751	0.175	0.847
FSW	0.113	0.977	0.025	0.969
FLW	0.790	0.560	-0.146	0.959
FLSWR	-0.950	-0.046	0.142	0.925
LL	0.373	0.241	-0.858	0.934
LW	0.762	0.536	0.140	0.887
LA	0.764	0.525	-0.180	0.891
E	-0.899	-0.353	0.029	0.934
LT	0.171	0.281	0.910	0.935
A	0.759	-0.506	0.017	0.833
gs	0.803	-0.445	0.166	0.870
Ci	0.866	0.075	0.039	0.756
Factor variance ratio %	49.85	29.47	11.22	90.55
